# Monte Carlo simulation and experimental validation of plant microtubules cathode in biodegradable battery

**DOI:** 10.1038/s41598-023-36902-x

**Published:** 2023-06-27

**Authors:** Kaushik A. Palicha, Pavithra Loganathan, V. Sudha, S. Harinipriya

**Affiliations:** 1 Research and Development Center, Ram Charan Co Pvt Ltd – Entity1, Chennai, Tamilnadu 600 002 India; 2 Department of Physics and Nanotechnology, SRMIST, Kattankulathur, Chennai, Tamilnadu 603203 India; 3 Department of Chemistry, SRMIST, Kattankulathur, Chennai, Tamilnadu 603203 India

**Keywords:** Biotechnology, Environmental sciences, Energy science and technology

## Abstract

For the first time, electrochemical methods are utilized to study the response of tubulin monomers (extracted from plant source such as Green Peas: Arachis Hypogea) towards charge perturbations in the form of conductivity, conformational changes via self-assembly and adsorption on Au surface. The obtained dimerization and surface adsorption energetics of the tubulins from Cyclic Voltammetry agree well with the literature value of 6.9 and 14.9 kCal/mol for lateral and longitudinal bond formation energy respectively. In addition to the effects of charge perturbations on change in structure, ionic and electronic conductivity of tubulin with increasing load are investigated and found to be 1.25 Sm^−1^ and 2.89 mSm^−1^ respectively. The electronic conductivity is 1.93 times higher than the literature value of 1.5 mSm^−1^, demonstrating the fact that the microtubules (dimer of tubulins, MTs) from plant source can be used as a semiconductor electrode material in energy conversion and storage applications. Thus, motivated by the Monte Carlo simulation and electrochemical results the MTs extracted from plant source are used as cathode material for energy storage device such as Bio-battery and the Galvanostatic Charge/Discharge studies are carried out in coin cell configuration. The configuration of the bio-battery cell is as follows: Al/CB//PP-1M KCl//MTs/SS; where SS and Al are used as current collectors for cathode and anode respectively, Polypropylene (PP) membrane soaked in 1M KCl as electrolyte and Carbon Black (CB) is the anode material. Another configuration of the cell would be replacement of CB by biopolymer such as ethyl cellulose anode (Al/EC/PP-1M KCl/MTs/SS).

## Introduction

**Figure 1:**
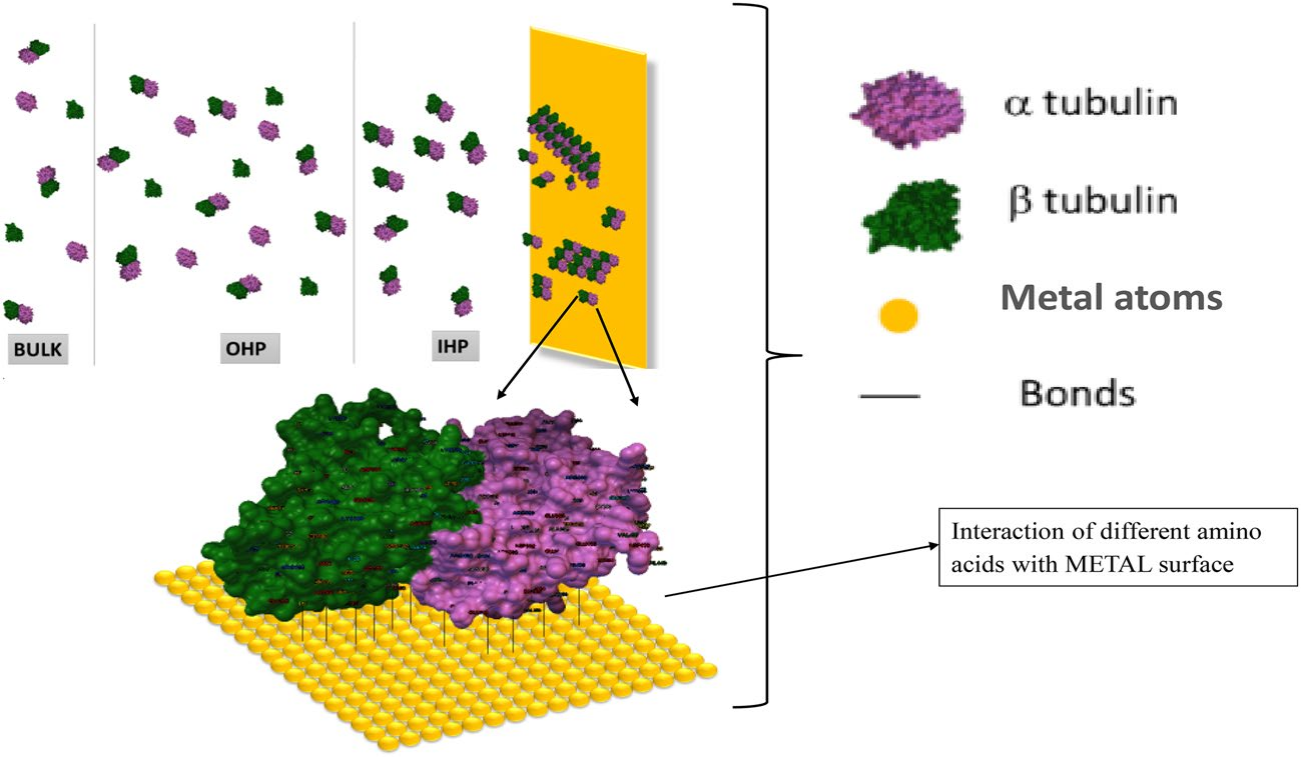
Interaction of tubulins forming MT on Au surface.

**Figure 2:**
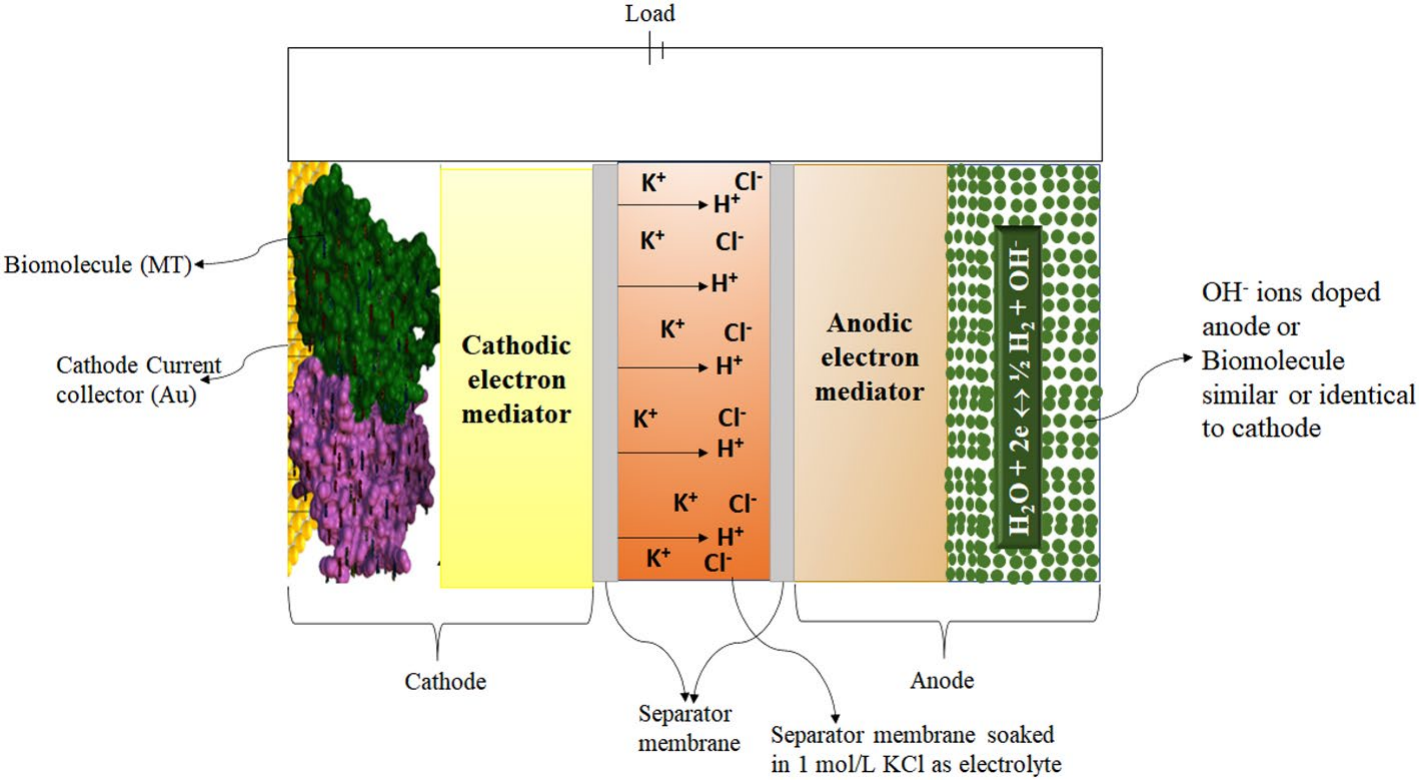
Illustrative configuration of Bio-Battery cell.

MTs are self-assembled polymeric tubules made of α and β tubulin dimers. MTs are the major constituent of cytoskeleton involved in variety of functions such as cell division, intracellular transport of organelles and prominently neuronal signal transmission^1–4^ due to their high ionic conductivity, unique structure and ordered arrangement^5^. Living cells are known to possess intrinsic electric fields and are sensitive to external electric fields. MTs are influenced by the external electric fields and thereby affect their major role in cell division. As cancer being an uncontrolled cell division^6–9^, MTs mechanism of action in treating cancers are also affected by electrical perturbations. It is found in the literature that protein monomers and their polymeric forms differ largely in their conductivity^5^. Hence proteins in the cells can be regarded as semiconducting in nature^10^. In the ionic medium inside the cell, counterion condensation effect compensates these unbalanced charges in polymeric forms of protein^11^. Hence studying the effects of electric field on the monomers and polymer ensembles of tubulin will provide insights in to the role of MTs in signal transmission. MTs are intracellular cytoskeletal cylindrical protein polymer made of tubulin dimer that self-assemble in to MTs under appropriate conditions but also rapidly depolymerize^12^. These proto filaments form hollow MTs of 15–25 nm diameters and lengths up to few mm, with almost 900 amino acid residues and measures 4 × 5 × 8 nm^3^^13^^.^ They are involved in many critical cellular functions such as cell division, intracellular transport and signal transduction^14^. MTs are associated with many proteins to perform various functions. Motor proteins such as kinesin and dynein use MTs as track to carry cargo such as neurotransmitters and deliver them^15^ (Figs. 1, 2).

MTs are also known to play important role in the functioning of neurons and the brain, hence are said to be responsible for cognition, memory and consciousness^16,17^. Consciousness is thought to be the emergent phenomena of highly complicated nested complex of neurons within the brain but if we look closer it is indeed due to the quantum effects of the MTs present in the neuronal cells as said in orchestrated OR theory^17–19^. The unit of information processing in brain is the tubulin protein and change in their conformational state^20,21^. They are known to increase the conductivity of electrolyte solutions by 23%^13^. This high conductivity can be attributed to the counterion polarization effect where the positive ions such as K^+^ and Ca^2+^ adsorb on the highly localized negatively charged surface of MTs and due to the mobility of these ions along the length of the MT, the conductivity increases^22,23^. They also transmit signals received from the external environment into the cell through the initiation of signal transduction cascade^14^. Transmission of signal in terms of passage of charged particles such as electron and ions through them is possible because of the nanopores and defects present in their wall^24^. Cytoskeletal proteins transmit signals in the form of ionic solitons^25^. Bunch of MTs form bundles in neuronal cells act as bio-electrical transistor to generate electrical signals similar to action potential^16^. Analogously, here we use electrochemical techniques such as CV as a probe to study the changes in the conformation and conductivity of tubulin protein due to application of charge perturbations. The novelty of this work is that unlike literature^10,26–28^, in which conductivity of MTs were studied using non-electrochemical methods, we use electrochemical methods to study the tubulin monomers instead of MTs and their response to charge perturbations in terms of conductivity and conformational changes through self-assembly and adsorption on Au surface. The free energy of dimerization and surface adsorption were computed by Monte Carlo Simulation and compared with experimental free energy calculated from CV. Coin Cell of configurations Al/CB//1M KCl-PP//MTs/SS and Al/EC//1M KCl-PP//MTs/SS were fabricated and their Galvanostatic Charge/discharge profile and performance is demonstrated.

## Methodology

The methodology is to develop general theoretical method to simulate the dynamic behavior of MTs under wide range of conditions similar to experimental conditions like charge perturbations by CV experiments and to validate the theory. From CV, using Randle Sevcik’s Equaion^29^, the diffusion coefficient of K^+^ ions in the MT is evaluated. Employing Butler–Volmer equation, the free energy of dynamic instability of MTs is obtained. The exchange current density is written as^29^

1 $$i_{0} = \frac{{nFC_{MT} K_{het} }}{A}{\text{exp}}\left( {\frac{{\beta nFE_{MTinitial/MTfinal} }}{{k_{b} T}}} \right)$$

and the heterogeneous rate constant is written via free energy of activation using Arrhenius equation as^29^

2 $$k_{het} = \frac{{k_{b} T}}{h}{\text{exp}}\left( { - \frac{{\Delta G_{act}^{ \ne } }}{{k_{b} T}}} \right)$$

**Figure 3:**
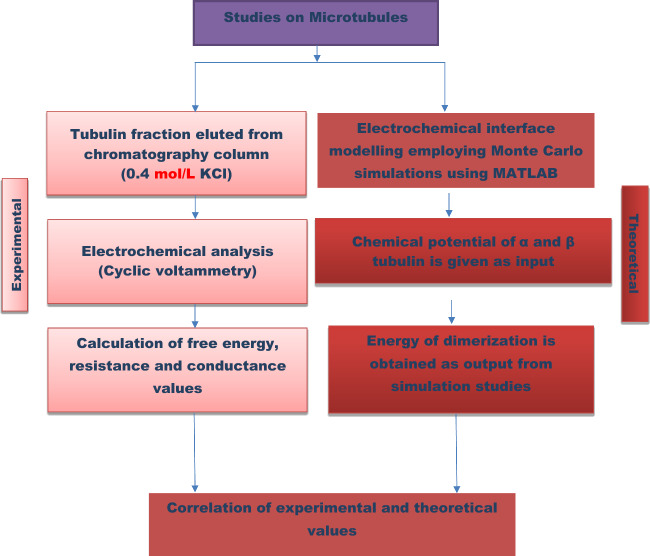
Flow chart of the methodology followed in the present work.

In case of charge perturbation, potential or current (potentiostatic or galvanostatic) is provided as input for MC simulation and the free energy of activation for dynamic instability of MTs in the presence of external perturbation were investigated. The comparison of conductivity (electronic and ionic), between theoretical and experimental procedures can also be made in addition to free energy estimation. The methodology is provided as flow chart in Fig. 3.

## Experimental section

As demonstrated elsewhere^30^, MT is isolated from plant source Arachis Hypogea. The column chromatography eluent is used in KCl electrolyte for electrochemical studies. All experiments were carried out in STP conditions. All the experimental research and field studies on plants (either cultivated or wild), including the collection of plant material, complies with relevant institutional, national, and international guidelines and legislation. All methods were performed in accordance with the relevant guidelines/regulations/legislation. The source of the plant material is as follows:

Nature of Biological resource: Plant.

Common Name: Peanut, Groundnut.

Scientific Name (Genus and Species) : Arachis Hypogea.

Exact part used: Seed.

Source of access (Wild/Culture/Trader): Trader.

Exact place (village, Taluk, District, State) of access of Biological source: Farmers Market, High road, Chengalpattu, Chengalpattu District, Tamil Nadu, India, 603001.

### CV studies

The CV studies were performed for the tubulin fraction obtained from the column chromatography in 0.4 mol/L KCl solution. The electrodes used were (i) Au—working electrode, (ii) Pt wire—counter electrode and (iii) Ag/AgCl—reference electrode employing Zahner-Zennium workstation (Germany). The Area of the electrode exposed to the electrolyte is 0.0005 m^2^. CV is recorded at a scan rate of 10, 20, 50, 100, 150 and 200 mV**/**s in the potential window of -1 to 1 V.

### Characterization of coin cell Bio-batteries with microtubules as cathode material

The electrochemical characterization of the coin cell of bio-battery fabricated using microtubules as cathode active material coated on SS current collector, carbon black or ethyl cellulose as anode active material coated on Al current collector, PP as membrane soaked in 1 mol/L KCl solution as electrolyte was subjected to Galvanostatic Charge Discharge (GCD) studies employing Zahner Zennium E4 Electrochemical workstation at 0.1 C-rate with the discharge current of 10 mA upto 10,000 cycles.

## Theoretical studies

**Figure 4:**
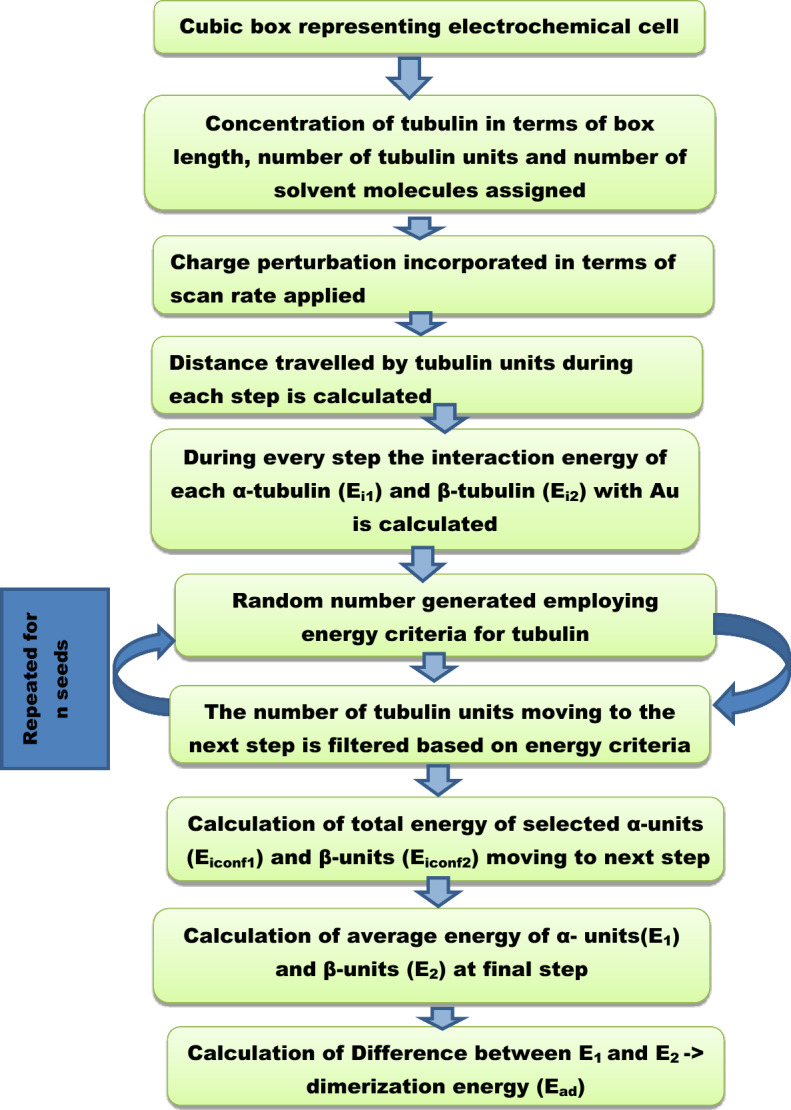
Flow chart of Monte Carlo Modelling of Electrochemical interface.

MC code was written using MATLAB software (version 7.5) in which *scan rate*, distance from the bulk to the electrode surface, chemical potential of α and β tubulin, number of amino acids present in the dimer (N) were provided as input. The random number generator and energy criteria were used to filter out the tubulin which possessed the energy required to move to the next step. The distance from the bulk to electrode surface and number of solvent molecules were fixed and several iterations from 10 to 10^5^ seeds were carried out for convergence. Using the model, energy associated with two configurations α and β tubulin is generated as output. Free energy (ΔG) was calculated as the difference between the energetics of the two configurations of tubulin. This energy difference between the two forms of tubulin is defined as the energy of dimerization of the tubulin monomers. The steps involved in the simulation is provided in Fig. 4. The MC simulation is carried out in STP conditions.

## Results and discussion

The energetics obtained from MC simulation and CV experiments for α and β Tubulin were analyzed and compared in the following sections.

### CV analysis

**Figure 5:**
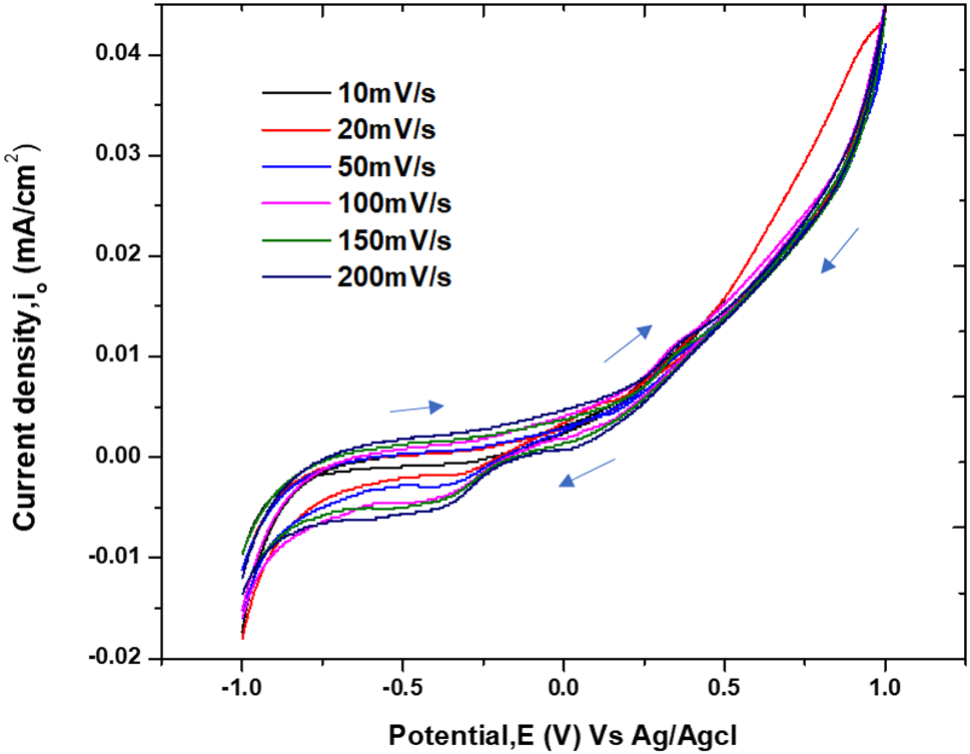
CV of MT eluent in 0.4 mol/L KCl at different scan rates. The arrows indicate the direction of scan rates.

**Figure 6:**
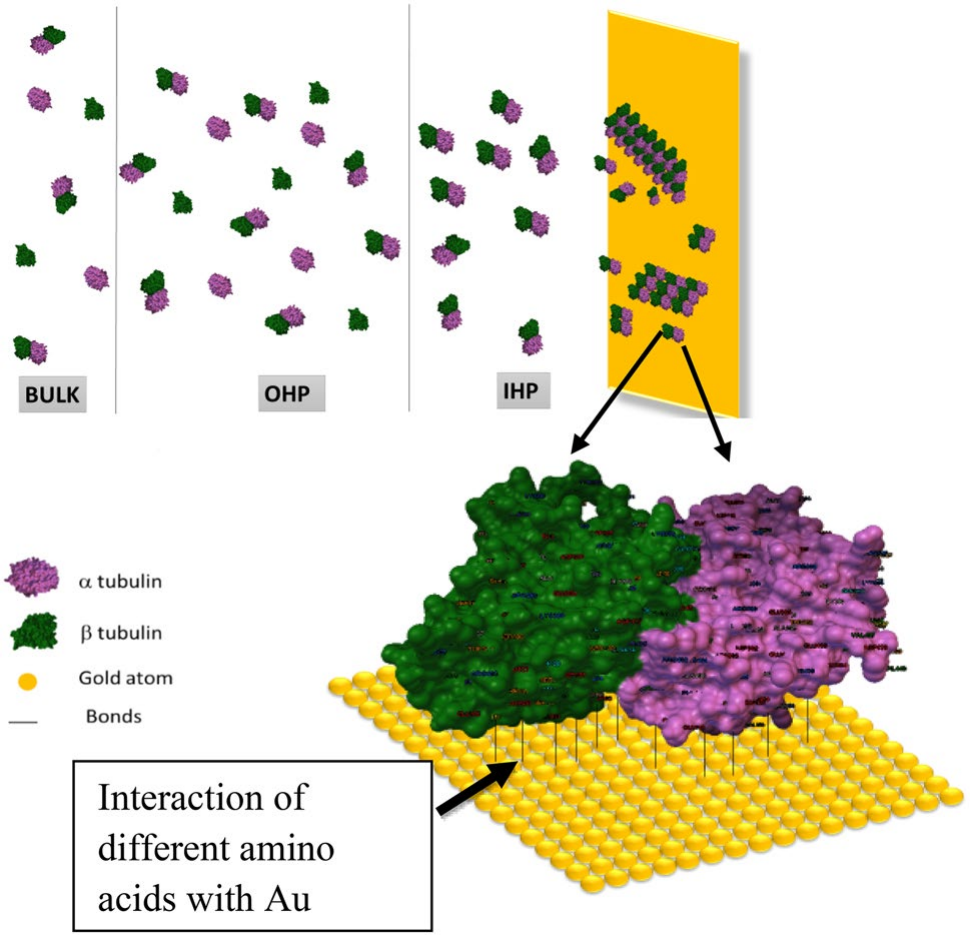
Illustrative representation of the electrode/electrolyte interface and the interaction between the protein units and the gold surface.

The eluent from column is utilized for electrochemical studies in 0.4 mol/L KCl. From Fig. 5, we can infer that at all the scan rates (or load applied), forward scan involved a small peak at 0.3 V triggered by the adsorption of MTs from the solution on Au surface. In the reverse scan, peaks at 0.05 and − 0.3 V were noticed. The former peak could be attributed to the Hydrogen Evolution Reaction (HER)^31^ whereas the latter one is due to desorption of MTs from the Au surface to the electrolyte solution as can be visualized in Fig. 5. The reversible nature of 0.3 V peak indicates that the adsorbed MT comes back to the electrolyte without any degradation on Au surface. As the applied load increases, the tubulin adsorption process becomes more facile because of the decrease in activation energy (cf. “Free energy of adsorption of MT on Au surface (ΔG) calculated from CV” section). As sulphur has natural affinity towards Au, sulphur containing amino acids such as cysetine and methionine present in tubulin interacts with the Au electrode via Au–S bond^32^*.* The first image (I) in Fig. 6 shows the electrochemical interface with the electrolytic solution containing the individual tubulin units forming dimers and diffusing towards the electrode eventually covering its surface with proto-filament chains. The second image (II) of Fig. 6 shows the interaction between the amino acids such as cysteine, methionine, phenylalanine, tyrosine and tryptophan residues of the protein chain with the Au surface.

### Free energy of adsorption of MT on Au surface ($\Delta {\varvec{G}}$ ) calculated from CV

**Figure 7:**
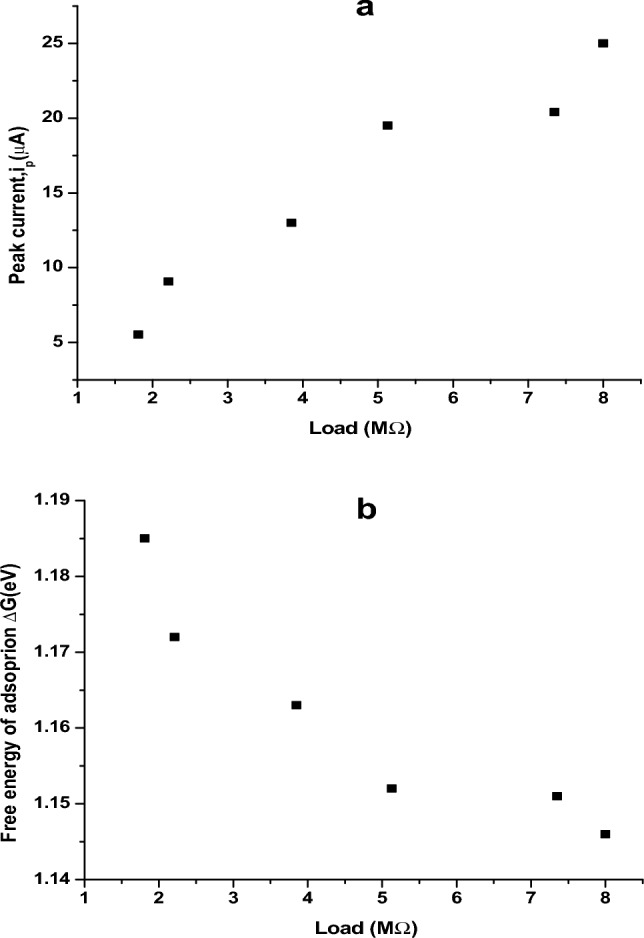
Variation of (**a**) peak current (i_p_) and (**b**) free energy of adsorption (ΔG) with increasing Load

The Free energy of adsorption of MT on Au surface ($\Delta {\varvec{G}}$ ) can be calculated via Butler-Volmer kinetics. $\Delta {\varvec{G}}$ is related to i_p_ via the following equations

3 $$\Delta {\varvec{G}} = \ln \left[ {\frac{{i_{o} Ah}}{{nFk_{B} TC}}} \right]RT$$

4 $$i_{o} = \frac{{i_{p} }}{A}$$

where *i*_*p*_ denotes peak current from CV in Amps, A is the area of electrode in cm^2^, h represents planks constant (Js), k_B_ depicts Boltzmann constant in JK^−1^, T is the temperature (298 K), C being the concentration of MTs in mol/cm^3^ and R represents the gas constant (Jk^−1^ mol^−1^). From CV, *i*_*p*_ at different load is utilized to evaluate the corresponding $\Delta \mathbf{G}$ as provided in Table 1. As the load increases, *i*_*p*_ increases by 5 times and $\Delta \mathbf{G}$ decreases by 40 meV (cf. Figure 7). This energy is nearly equivalent to free energy of tubulin immobilization^33^ (50 meV) on MTs, which indicates the incorporation of tubulin (monomers) in MTs(polymer) with application of load. Thus, it could be inferred that with increased charge perturbation, the feasibility of MT being adsorbed on the Au surface increases. Since there is no degradation seen in CV analysis, $\Delta \mathbf{G}$ shows very minimal variation with the load applied (40 meV).

**Table 1:** Variation of i_p_, ΔG, ρ and σ with Load.

Scan rate (mV/s)	Load (MΩ)	Peak current, i_p_ (µA) (From CV data)	ΔG (eV)	R_ct_ (Ω)	Resistivity, ρ (Ω m)	Conductivity, σ (mS m^−1^)
10	1.81	− 5.52	1.19	2.33	1162.96	0.86
20	2.21	− 9.07	1.17	1.42	707.77	1.41
50	3.85	− 13.0	1.16	0.99	493.81	2.03
100	5.13	− 19.5	1.15	0.66	329.21	3.04
150	7.35	− 20.4	1.15	0.63	314.68	3.18
200	8.00	− 25.0	1.15	0.51	256.78	3.89

### Conductivity of MT from CV studies

The charge-transfer resistance (R_ct_) and electronic conductivity of tubulin dimers were calculated using the following Equations^34^ and the values are given in Table 1.

5 $$R_{CT} = {\raise0.7ex\hbox{${RT}$} \!\mathord{\left/ {\vphantom {{RT} {nFi_{o} }}}\right.\kern-0pt} \!\lower0.7ex\hbox{${nFi_{o} }$}}$$

6 $$\uprho = {\raise0.7ex\hbox{${ R_{CT} A}$} \!\mathord{\left/ {\vphantom {{ R_{CT} A} l}}\right.\kern-0pt} \!\lower0.7ex\hbox{$l$}}$$

7 $$\upsigma = {\raise0.7ex\hbox{$1$} \!\mathord{\left/ {\vphantom {1 \rho }}\right.\kern-0pt} \!\lower0.7ex\hbox{$\rho $}}$$

It is found that with increase in applied load, i_p_ increases indicating a linear proportionality between the load and the intensity of peak current. From Table 1, it had been observed that the resistivity (ρ) of the dimers decreased linearly with increasing load. The electronic conductivity (σ) showed an exponential increase with increasing load. Hence it is concluded that as the magnitude of the voltage applied increases, the resistance and the free energy of interaction of the dimers decreases, thus they become more reactive to their surroundings by taking up the charged particles around them and conducting the positive ions such as K^+^ from bulk to the Au surface.

### Dynamic stability of MTs on Au surface

**Figure 8:**
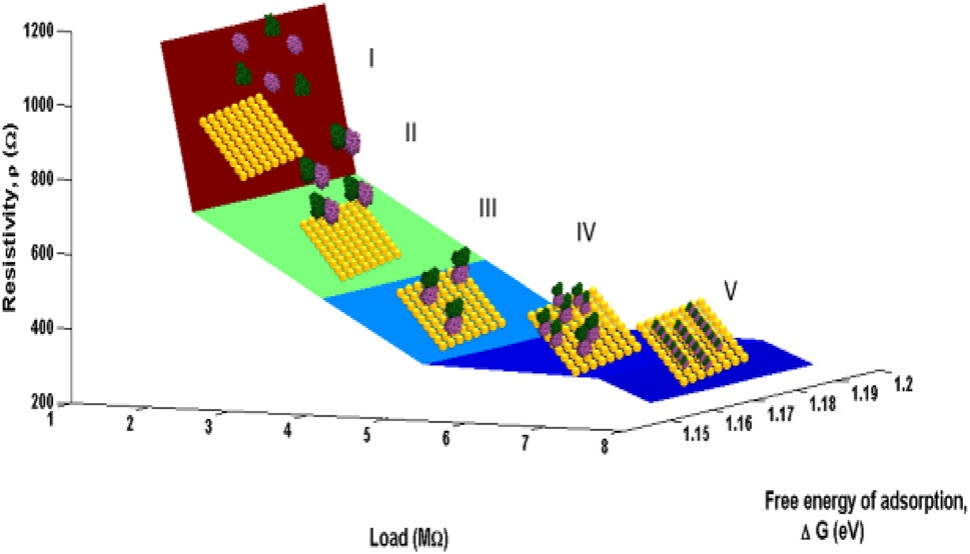
Dynamic stability of MTs on Au surface.

**Figure 9:**
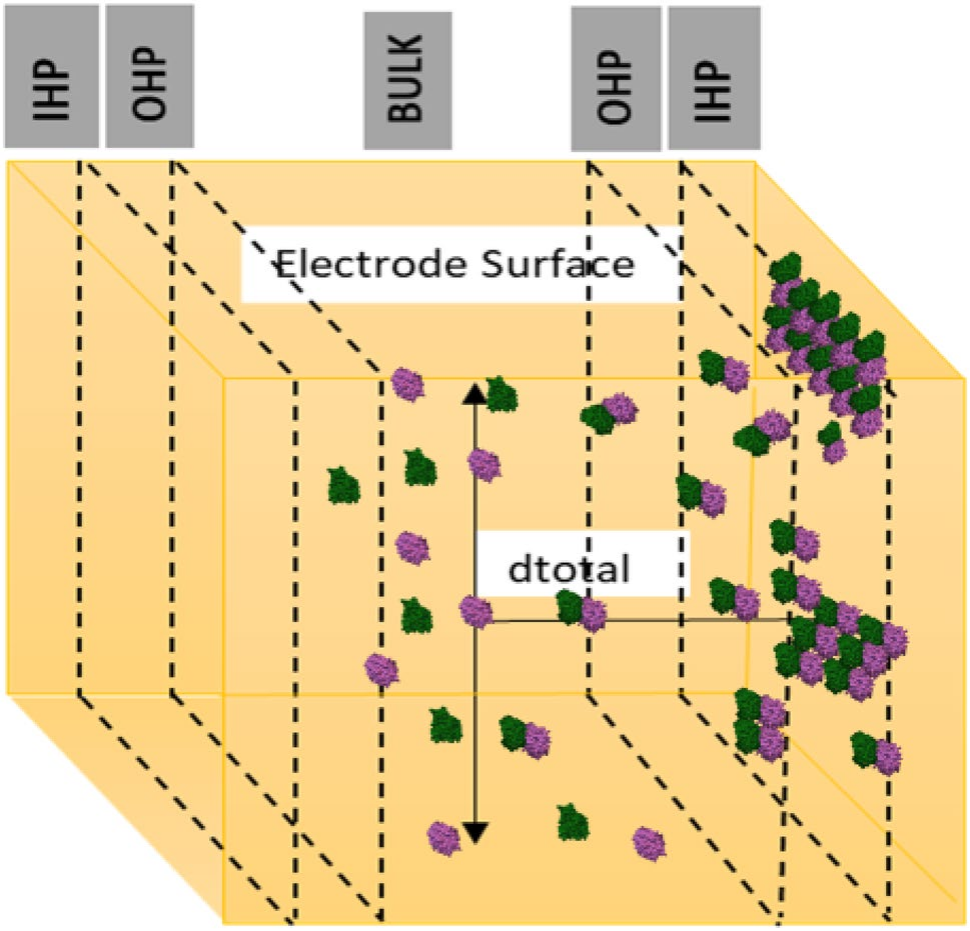
Simulation box mimicking the electrode/electrolyte interface.

Figure 8 shows the evolution of **ΔG** during the transfer of tubulin monomers to Au surface from the bulk of the solution. Steps (I–V) indicate processes by which the tubulin units diffuse towards the Au surface. As observed, upon experiencing higher load in MΩ, a decreasing trend in the ΔG with applied load indicates facile dimerization of α and β tubulin monomers into MTs. Initially the monomer units coexisted without strong interaction between them (I). As the load applied per second increases, ΔG slowly decreases with the α and β-tubulin interacting to form dimers (II) via electrostatic interaction, H-bond formation and vanderwaals forces^35^. These dimers adsorb on Au surface due to the interaction between amino acid residues in tubulin such as cysteine, methionine, tyrosine and Au surface. The interaction between Au and tyrosine, tryptophan is through conjugated π- electrons and in the case of cysteine and methionine via hetero sulphur atoms^32^ as represented in Fig. 8. Many such dimers start covering the electrode (Au) surface (IV) and due to the crowding of dimer units, they associate themselves as self-assembly to form linear polymer filaments (V) and attain stability. As the applied load increases, decrease in ρ (increase in conductivity σ) with simultaneous decrease in ΔG had been noticed. This could be attributed to the self-assembly of monomers forming dimers and the dimers interacting with Au surface. Free monomers in the solution have high energy and when they assemble in to ensembles this energy reduces. In the case of free monomers, the electronic band gap between the conduction and valence band is large and when they start aggregating in an orderly manner, this gap decreases^5^, making the conductivity (σ) to increase as supported by decrease in resistivity (ρ) in Fig. 8. Conductivity (σ) measurements of several proteins under various conditions indicated a band gap or activation energy of the order of 3 eV^10,36,37^. Thus, from Fig. 8 which represents the phase transition in MT due to charge perturbations, the maximum limit of ΔG is found to be 1.22 eV and is in satisfactory agreement with the dimerization energy (1.10 eV) of tubulin monomers^27,28^. The difference in ΔG with respect to the difference in load is nearly 40 meV, such small variation may be due to the conformational change of independent α and β tubulin subunits to α, β-dimer and their incorporation into MT^33^. Thus Fig. 8, clearly indicates the dimerization of tubulin monomers, its self-assembly into linear proto filaments and adsorbing on Au surface with increase in load applied. It could also be inferred that electronic conductivity (σ) of tubulin monomers (dimerizing and self-assembling) increased upon transfer from bulk to Au/electrolyte interface. The electronic conductivity (σ) evaluated from CV studies is of the order of 0.86 to 3.89 mSm^−1^ demonstrating the increase in electrical conductivity (σ) by 4.58 times for 20 times increase in the load. This supported the fact that proteins can be semiconducting in nature^10^, especially MTs and can be of great importance in bioelectronic applications such as biodegradable batteries, pace makers so on and so forth. In addition, Figs. 8 and 9, explicitly depicts the dynamic nature of the MTs formed on Au surface to the self-assembly of tubulin monomers in bulk of the electrolyte upon charge perturbations.

### MC simulation analysis

In order to support the experimental data and to get a clear insight about the interfacial reactions, the adsorption of the tubulin proteins on Au surface was modelled using MC simulation.

**Figure 10:**
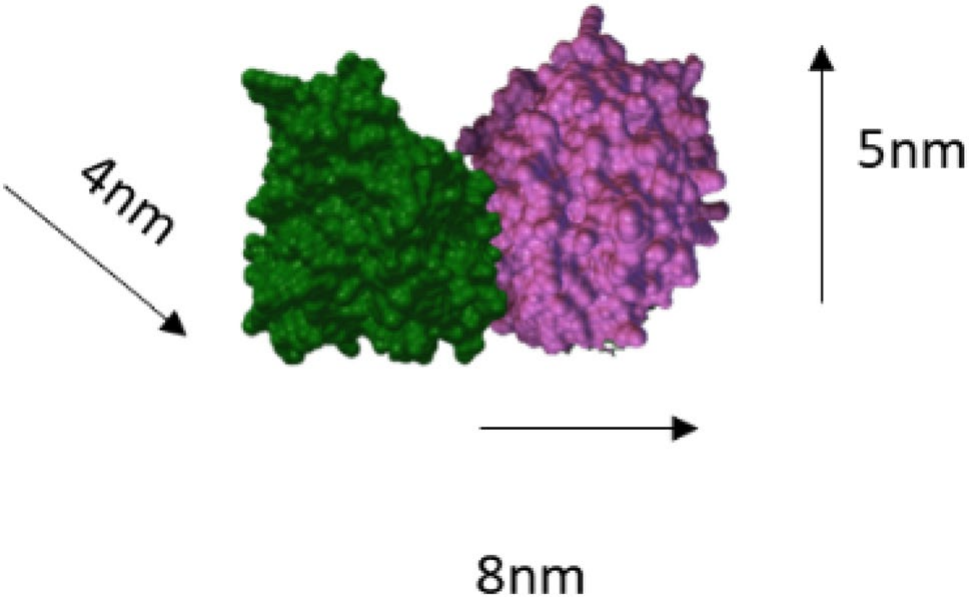
Tubulin dimer of 4 nm × 5 nm × 8 nm.

**Figure 11:**
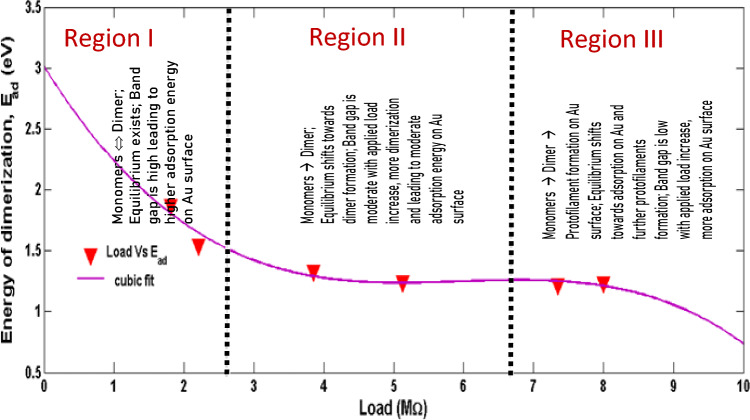
Variation of E_ad_ with Load.

Assumptions made:

- Simulation box walls were assumed to be the surface of the electrode (Au)
- The distance from the center of the box to the walls is maintained constant; *d*_*total*_ = 690 Å
- Number of protein units was increased from 1 to 1500; *a* = 1 to 1500
- With increasing tubulin units, the concentration per unit area increases.
- Figure 9 is the analogous representation of electrode surface, Inner Helmholtz Plane (IHP), Outer Helmholtz Plane (OHP) and bulk of the electrolyte, whereas when the protein moves from bulk to the metal surface, its concentration decreases.
- Figure 9, mimics the electrochemical interface and bulk of the solution with protein units in the center (bulk) and they move from the center to the edge of the box (electrode surface)
- The iteration continues till the distance between the protein units and the electrode surface (the walls of the box) becomes less than the critical length which is assumed in the present scenario as the Au–S bond distance, d_1_.
  $$while \, d_{total} \ge d_{1}$$
- The energetics involved in each step movement of the protein units inside the cubic box is calculated using Eqs. (8–15).
- The difference in the energy associated with the movement of α and β tubulin is calculated to be the energy of dimerization of tubulins on Au surface into MTs (Fig. 10)
- The tubulin unit dimensions are 4 × 5 × 8 nm^3^ as represented in Fig. 11.

#### Adsorption energetics of MT-Au from MC analysis

A model was developed using MATLAB in which the simulation box is a cube, whose side length is considered as 2 × d_total_. This value is fixed as 690 Å. The center of the box is assumed to be the bulk of the electrolyte and walls are the electrode surface. The number of tubulin units and the water molecules are assigned as input. Random number is generated employing the criteria

$$ir\left( j \right) \, \ge e^{{ - \left( {F\left( {\mu_{2} - \mu_{1} } \right)/RT} \right)}}$$

where F represents Faraday constant (C/mol), µ_1_ and µ_2_ denotes the chemical potential (eV) of α and β tubulin units respectively, R being the Gas constant (J/K/mol) and T is the working temperature in K. The tubulin units were allowed to move from the center of the box to the walls, the distance covered by a single tubulin unit during each step (**d**_**2**_) is given by the following equation

8 $${\varvec{d}}_{{\mathbf{2}}} = {\varvec{d}}_{1} + \Delta {\varvec{d}}$$

9 $$\Delta {\varvec{d}} = {\varvec{x}}*\frac{t}{{t_{eq} }}$$

where **x** is the hydrodynamic radius/solvation radius of tubulin monomer in the electrolyte (3 Å), $\Delta {\varvec{d}}$ is the distance covered in each step, t is the time scale of applied potential (*ephiapp*), t_eq_ is the equilibration or relaxation time between one step and another step, d_1_ is the Au–S bond distance (2.39 Å)^36^. The product of solvation radius of each tubulin along with the time fraction will give us the distance that can be covered during single step. Using MC simulation, the energy values assigned are used to filter the tubulin units which has energy value above or equal to a critical energy value employing Arrhenius equation as criteria; (${e}^{-\left(F\left({\mu }_{2}-{\mu }_{1}\right)/RT\right)})$, those tubulin units which possess this minimum energy will move to next step. This process is repeated for many iteration/seeds to check reproducibility.µ_1_ and µ_2_; the chemical potential of α and β tubulin is defined as the energy possessed by them without the externally applied potential and is given by the below equations

10 $${\upmu }_{1} = E_{i1} - ephiapp$$

11 $${\upmu }_{2} = E_{i2} - ephiapp$$

where ${E}_{i1}$ and ${E}_{i2}$ are energy associated with the two configurations of tubulin given by the sum of individual α/β energy, distance fraction and miscellaneous energy as given below

12 $$E_{i1} = \left( {E_{amt} + \left( {\frac{d2}{{d1}}} \right)} \right) + E_{mis}$$

13 $$E_{i2 } = \left( {E_{bmt} + \left( {\frac{d2}{{d1}}} \right)} \right) + E_{mis}$$

E_amt_ and E_bmt_ represents the interaction free energy of α-tubulin and β-tubulin with Au surface respectively (E_amt_ = 0.286 eV, E_bmt_ = 0.293 eV)^32^

The miscellaneous energy is the solvation energy of the tubulin units, i.e., H-bonding and is given by the below equation as the sum of distance covered and applied potential over the total box size

14 $$E_{mis} = \left( {d + ephiapp} \right)/d_{total}$$

The difference between *E*_*i1*_ and *E*_*i2*_ is considered as energy of dimerization, *E*_*ad*_

15 $$E_{ad} = \, E_{i1} - E_{i2}$$

The process is repeated for 10^3^ to 10^5^ seeds to attain convergence and reproducibility (cf. Figure 2).

#### Variation of E_ad_ with applied load

From Fig. 10, it is observed that with increase in the applied load an exponential decrease in the dimerization energy (*E*_*ad*_) is observed in the system. Initially at lower load, the relaxation time for the tubulin proteins is very high between any applied potential at any instant. Thus the equilibrium between dimerization and de-dimerization process happens at faster rate and hence the higher barrier for dimerization. While as the load increased, high potential pulse is applied per second, dimerization happens instantly without de-dimerizing. Thus, the energy barrier for dimerization diminishes and hence the dimer formation become feasible. In addition, with increasing load, the monomer units are driven more towards the electrode surface, which makes the dimerization and subsequent adsorption process more facile and highly feasible. Figure 10 shows the load dependency of *E*_*ad*,_ as the load applied increases, the *E*_*ad*_ value decreases, which leads to the conclusion that applied load makes the adsorption of monomer units on the Au surface and dimerization easier which is evident from the exponential drop in the dimerization energy with increasing load. As the voltage applied per second is increased in our model the mobility of monomers towards the Au surface increases and dependency is given by the equation

16 $${\text{E}}_{{{\text{ad}}}} = \left( {8.9*10^{ - 21} } \right){\text{ L}}^{3} + \, \left( {1.6*10^{ - 13} } \right){\text{ L}}^{2} {-} \, \left( {8.3*10^{ - 7} } \right){\text{ L }} + \, 3.0$$

This equation signifies that in the absence of load (L → 0), the energy of dimerization is 3.0 eV. This is in good agreement with the experimental values of 2.6–3.1 eV found by Eley et al.^37^ and is approximately equivalent to the *forbidden band width between the highest filled and the unfilled band calculated using a simple (Hückel type) LCAO MO method (3.05 eV) in proteins stating the possibility of conduction in proteins like semiconductors*^38–40^.

#### Comparison of ΔG from CV and E_ad_ from MC simulation

**Figure 12:**
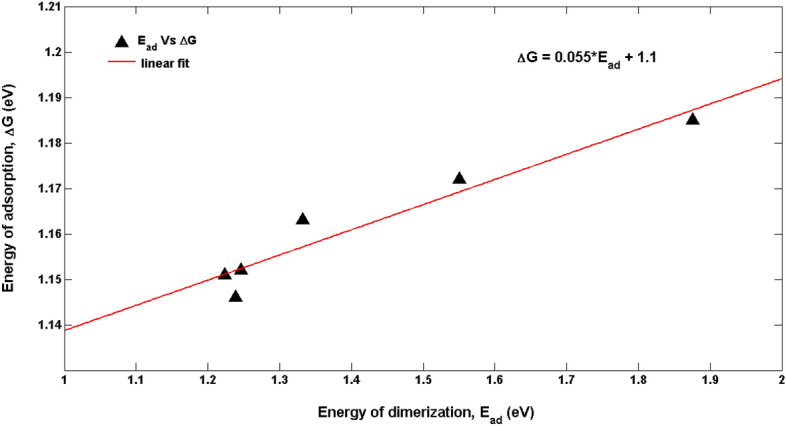
Plot between experimental ΔG and theoretical E_ad_.

**Figure 13:**
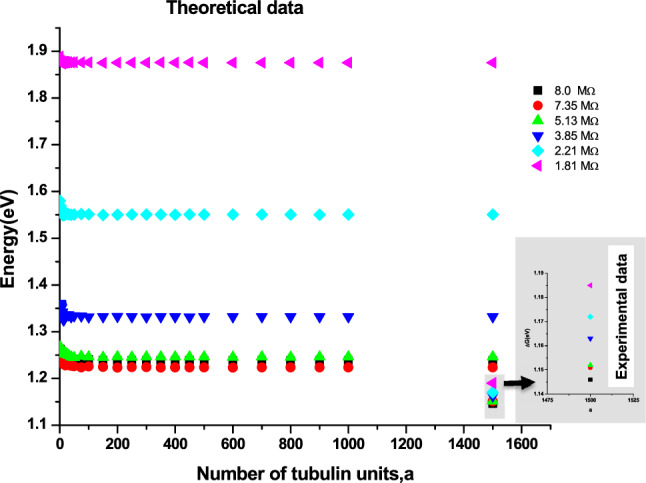
Variation of experimentally (∆G) and theoretically (E_ad_) calculated energy with Load.

**Figure 14:**
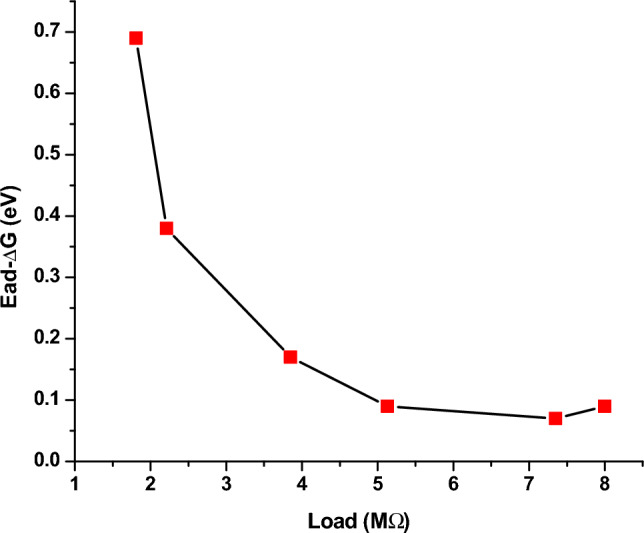
Variation between Theoretical and experimental energy with Load.

**Figure 15:**
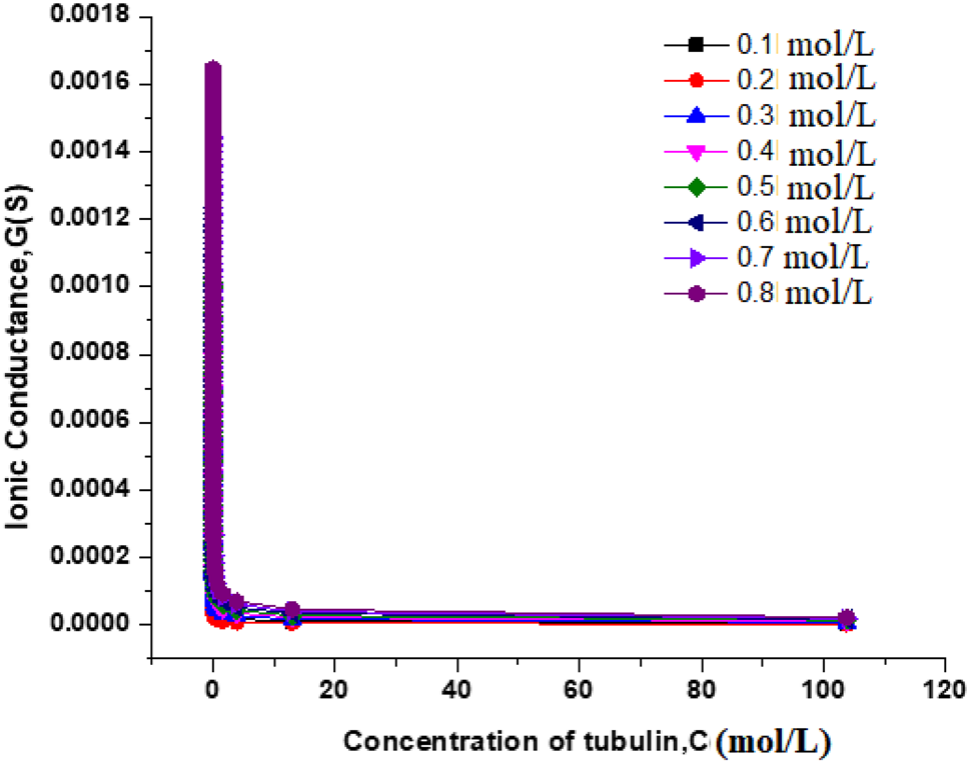
Ionic Conductance versus Concentration of MT at different concentrations of KCl.

For the concentration of 1 mmol/L MTs, experimentally obtained ΔG and the theoretically calculated dimerization energy *E*_*ad*_ were compared as shown in Fig. 12. It was found to be linear with a slope of 0.055 and an intercept of 1.10. This signifies that for every 1.0 eV of the dimerization energy there is a 0.055 eV change in the activation energy. The intercept signifies that in the absence of any dimerization process happening in the system (*E*_*ad*_ → 0); the average activation energy of tubulin would be 1.10 eV. It is shown in literature^41^ that the contribution of interfacial hydrogen bonding to the free energy of MT formation from α and β lattices as 462 kJ/mol (4.80 eV). Thus, the activation energy of 1.10 eV being comparatively lesser due to the conditions of the electrochemical setup, solvent, external electric fields and accounts only for the dimerization of α and β tubulin. Upon comparing the theoretical and experimental energetics, it can be inferred that there is exponential variation in the energetics with change in load. As the load increases, the rate of the electron transfer reaction at the Au surface takes place efficiently due to decrease in the energetics as indicated by the exponential decay in Fig. 12. From Fig. 12, it is seen that the experimental value is lower than the theoretical value. The justification behind this higher theoretical value could be attributed to the absence of ascribing parameters for dissociation of tubulin dimers on the Au surface in the MC simulation. It is also observed from Fig. 11 that as the load increases the theoretical and experimental values converge. This convergence demonstrates the diminishing of degradation of the tubulin dimers on the Au surface with increase in the load. The rationale behind this is the fact that, relaxation time of dimers between two impulse of potentials per second is very low at higher load conditions and hence at any instant, the dimers formed on Au surface gets involved in the formation of linear protofilament of MT rather than undergoing dissociation. With increase in the number of tubulin units in the simulation box, exponential decay of the energetics is noticed for all load. The concentration of extracted tubulin in the experiments is 1 mM (a = 1500 units), at that number of units (cf. Fig. 13) the experimental energetics was 1.10 eV but the theoretical values were higher. It is clear from Table 2 that at load of 1.81 to 2.21 MΩ, the energy difference between theoretical and experimental value ranges from 0.69 to 0.38 eV respectively. As can be verified by the literature, the difference in energetics could be attributed to the longitudinal free energy of dissociation of MTs (ca. 0.64 eV)^27^. This difference in energy varies for different loads and this additional energy in theoretical value is due to the fact that the dimerization energy is higher than the activation energy of tubulin and also the linear protofilament formation of tubulin dimers at higher loads. As the load increases, we observe an exponential decrease in the activation energy and dimerization energy, this is due to the interaction between monomers leading to the formation of aggregates such as dimers and oligomers while being incorporated into MT^33^, which is clear from the rate of energy drop (40 meV). Initially in their free form when the units exists as monomers the energy values are high. With increase in applied load, this energy drops indicating decrease in activation energy which leads to facile interaction between the monomer units and also facilitates their adsorption on the electrode surface. This decreasing trend in energy is seen both in experimental and theoretical results. This variation in energy is an indication of the process occurring at the interface. Thus, it is clear from Fig. 14, that the monomers are forming dimers, oligomers and MT ensembles and this led to a drop in energy. The dimerization energy calculated from MC simulation (*E*_*ad*_) and the activation energy calculated from CV(∆G) are compared and the difference between them is plotted with increasing load in Fig. 15 and provided in Table 2. It is seen that the difference reduces with applied load indicating a convergence between these two values and this difference can be attributed to the latitudinal (0.3 eV)^27^ and longitudinal (0.64 eV)^27^ free energy of dissociation of tubulin-tubulin bonds in MTs.

**Table 2:** Variation of Diffusion coefficient and mobility of K^+^ ion with load.

Load(MΩ)	i_p_ (µA)	D × 10^–7^ (cm^2^/s)	µ_K+_ × 10^–6^ (cm^2^/Vs)
1.81	− 5.52	1.76	6.97
2.21	− 9.07	2.38	9.43
3.85	− 13.0	1.96	7.76
5.13	− 19.5	2.20	8.71
7.35	− 20.4	1.61	6.38
8.00	− 25.0	1.80	7.13

#### Ionic conductivity of the electrolytic solution from MC studies

**Figure 16:**
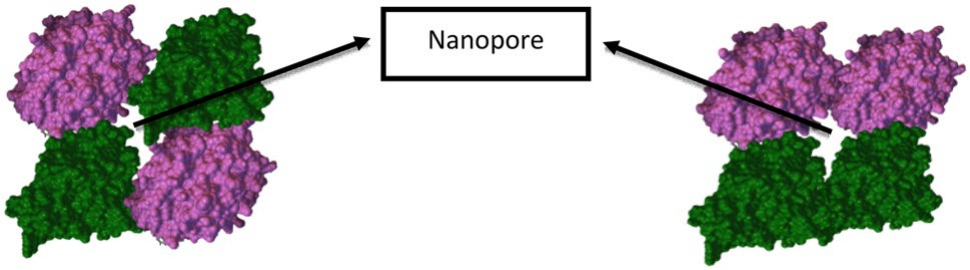
The nanopores formed between the α and β tubulin in both A lattice and B lattice MTs respectively.

Ionic conduction occurs in MTs due to two reasons (i) one being the highly negatively charged C terminal chain of the tubulin monomer and (ii) second being the pores between the tubulin dimers which has properties like ion channels^42,43^. The nanopores present in the walls of MTs helps them in passage of ions through the channels to the lumen of the hollow tubes (cf. Fig. 16). The pore size is big enough for K^+^ ions to pass through but not Ca^2+^ and Na^+^. This high negatively charged C terminal chain of the dimer is projected outside on the periphery such that the surface charge density is high and due to this counterions adsorb easily on the surface. The MT assembly consists of two types of lateral arrangement: (i) α-tubulin-α -tubulin, β-tubulin- β-tubulin ie α-tubulin/ β-tubulin in one protofilament lies beside its same counterpart in the neighboring protofilament (ii) α-tubulin-β-tubulin ie α-tubulin in one protofilament lies beside/β-tubulin in the neighboring protofilament and vice versa constituting the A lattice and B lattice respectively^44^. The ionic conductance of the electrolyte was calculated using the formula

17 $$G = \frac{{\Lambda_{{K^{ + } }}^{0} {\text{CA}}}}{l}$$

where G-Conductance of the electrolyte in mhos, ${{\Lambda }^{0}}_{{K}^{+}}$-Molar conductance of K^+^ in mhos, C—concentration of KCl in the electrolyte in mol/L, A—Area of the cross section of electrodes in cm^2^, l-distance between the electrodes in cm. The concentration of KCl is varied from 0.1 to 0.8 mol/L and the conductance is calculated for varying box dimensions (in turn concentration of MTs) and plotted as seen in Fig. 15. The molar ionic conductance of K^+^ ions is 73.5 S cm^2^/mol/L^45^ and the calculated value of ionic conductance for 1 mM tubulin at 0.4 mol/L KCl is 5.53 × 10^−4^ µS (ionic conductivity being 1.1 S m^−1^). It is seen that there is a sudden drop in conductance when the concentration of MTs reached a critical limit, with further increase in the concentration of MTs, the conductance becomes constant and independent of the concentration. This indicates that the individual tubulin units assemble into a particular conformation, till the critical point and the conductance decreases. After the attainment of critical or saturation point, conductance remains constant with variation in concentration. This behavior is observed for all concentrations of KCl. Analogous results had been reported in the literature, that with increase in the concentration of MT the conductance increases approximately by 0.2 mS up to 60 Hz of frequency of the AC signal and beyond this frequency the conductance starts to decrease and remains constant^11^ (Supplementary Information 1).

### Diffusion coefficient of the electrolyte from CV studies

Using Randle Sevcik’s Eq. ^29^ for quasi-reversible system the diffusion coefficient was calculated from i_p_

18 $$i_{p,f}^{irr } = \mp 0.446nFAC\sqrt {\frac{nfDv}{{RT}}}$$

The mobility of a species in electrolyte, K^+^ is directly proportional to the diffusion coefficient of that particular species and is given by Einstein–smoluchowski Equations^35^ as follows

19 $$\mu_{k + } = \frac{{\left| {\left. {z_{i} } \right|F} \right|D_{k + } }}{RT}$$

**Figure 17:**
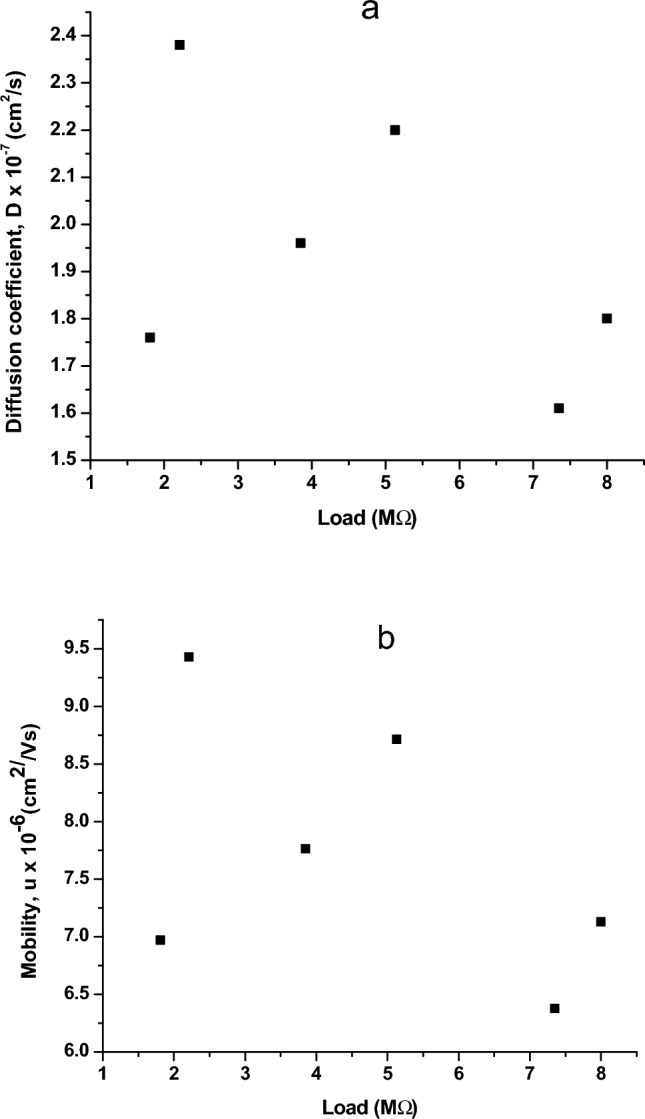
(**a**) Variation of Diffusion coefficient with Load (**b**) variation of mobility with Load.

Diffusion coefficient (D) from Randle Sevcik’s equation tells us about the extent of diffusion of analyte (tubulin) and its dependency on applied load. D is linearly proportional to the load in a diffusion limited process. Diffusion coefficient of tubulin in the electrolyte for varying load along with their mobility are given in Fig. 17. From Fig. 17, we infer that with increase in load, diffusion coefficient and mobility follow drastic pattern, increases and decreases with every load application, forming three spikes indicating the increased mobility of monomers in the electrolyte, followed by sudden drop. The reason behind this kind of variation is due to the shift in equilibrium between aggregated monomers (oligomers, dimers) and free monomer forms of tubulin. The aggregated tubulin has less mobility and D value whereas the free forms are able to move unhindered, hence showing a spike in the values of mobility and D, resulting in constant association and dissociation among themselves leading to dynamic instability. The average value of D is obtained as 1.95 × 10^–7^ cm^2^/s in agreement with the literature^46,47^ value of (4.5 ± 0.2) × 10^−7^ cm^2^/s for labelled tubulin at nano molar concentration. The calculated average value of mobility for tubulin is 7.73 × 10^–6^ cm^2^/Vs. Hence the electrochemical perturbations are clearly making the tubulin units associate and dissociate causing the rise and fall of Diffusion coefficient and mobility values with load.

## Galvanostatic charge/discharge profile of non-enzymatic bio-battery coin cell

Theoretical specific capacity of MTs is calculated from the formula.

Theoretical specific capacity = zF/M; where z is the charge, F is Faraday’s constant and M is the molecular weight of the material under consideration. In the present studies, charge of intercalation or deintercalation in the MTs would be one (K^+^ ions); F = 96,500 Cmol^−1^, the theoretical specific capacity of MTs were obtained as 89.35 mAh/g. For the charging current is 10 mA and discharging capacity is 89.35 mA, the C-rate obtained is 0.1 C.

**Figure 18:**
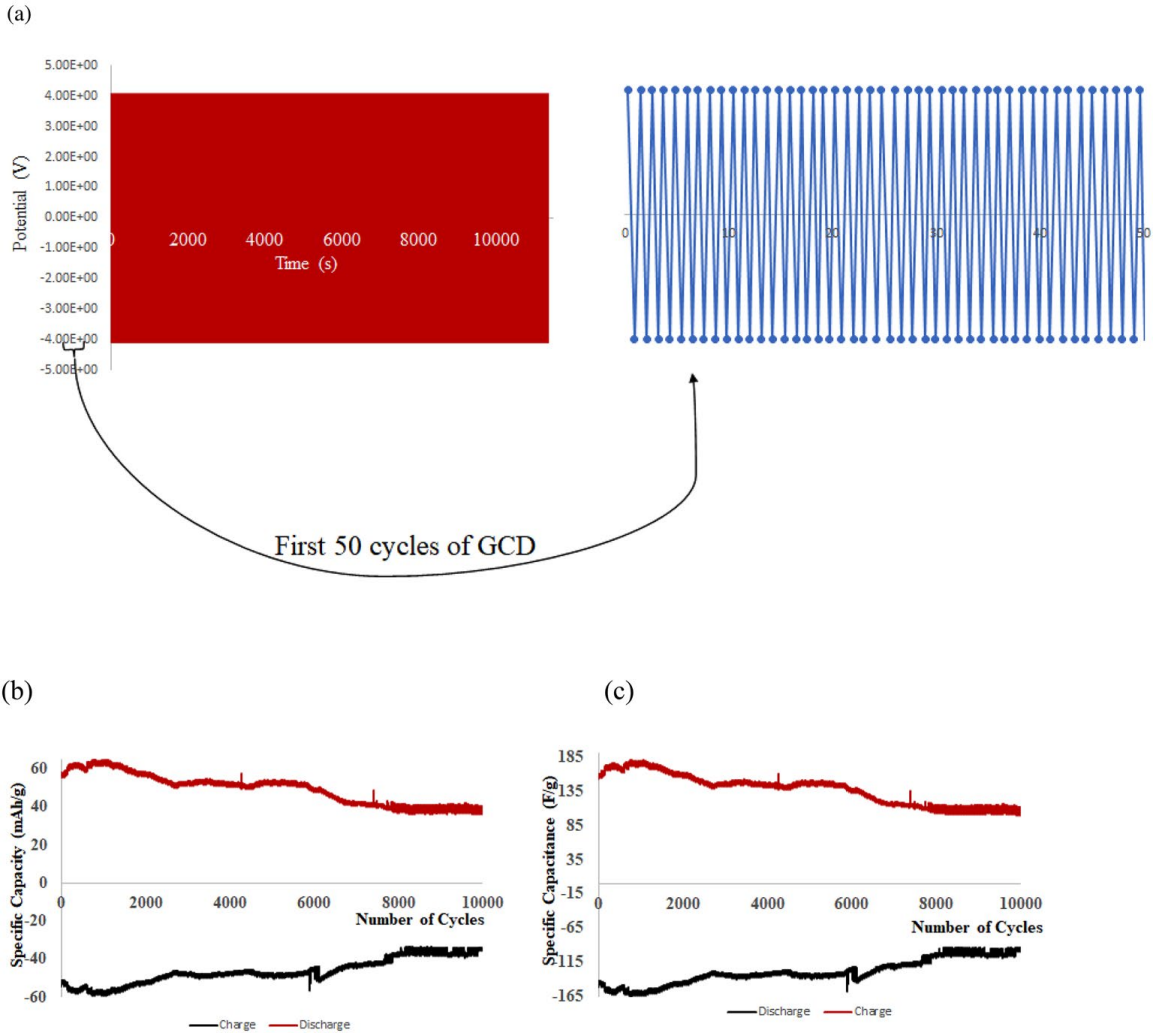
The (**a**) GCD curves, (**b**) Specific Capacity and (**c**) Capacitance profile of bio-battery cell of configuration Al/CB//PP-1 mol/L KCl//MT/SS with 10,000 cycles @ 0.1 C rate.

The GCD profile of the bio-battery coin cell is represented in Fig. 18a. The cycling of charge/discharge is carried out at 10 mA discharge current. The coin cell is stable up to 10,500 cycles and 175 h at 0.1 C-rate. The specific capacity of Non-enzymatic Bio-battery cell is calculated at every Δt(s) for the discharge current of I (Amps) from the GCD profile for the electroactive mass m (gms) as follows:

$${\text{Specific }}\,{\text{Capacity }} = {\text{ I}}\Delta {\text{t}}/{\text{m}}$$

Analogously,

$${\text{Specific }}\,{\text{Capacitance }} = {\text{ I}}\Delta {\text{t}}/{\text{m}}\Delta {\text{V}}$$

**Figure 19:**
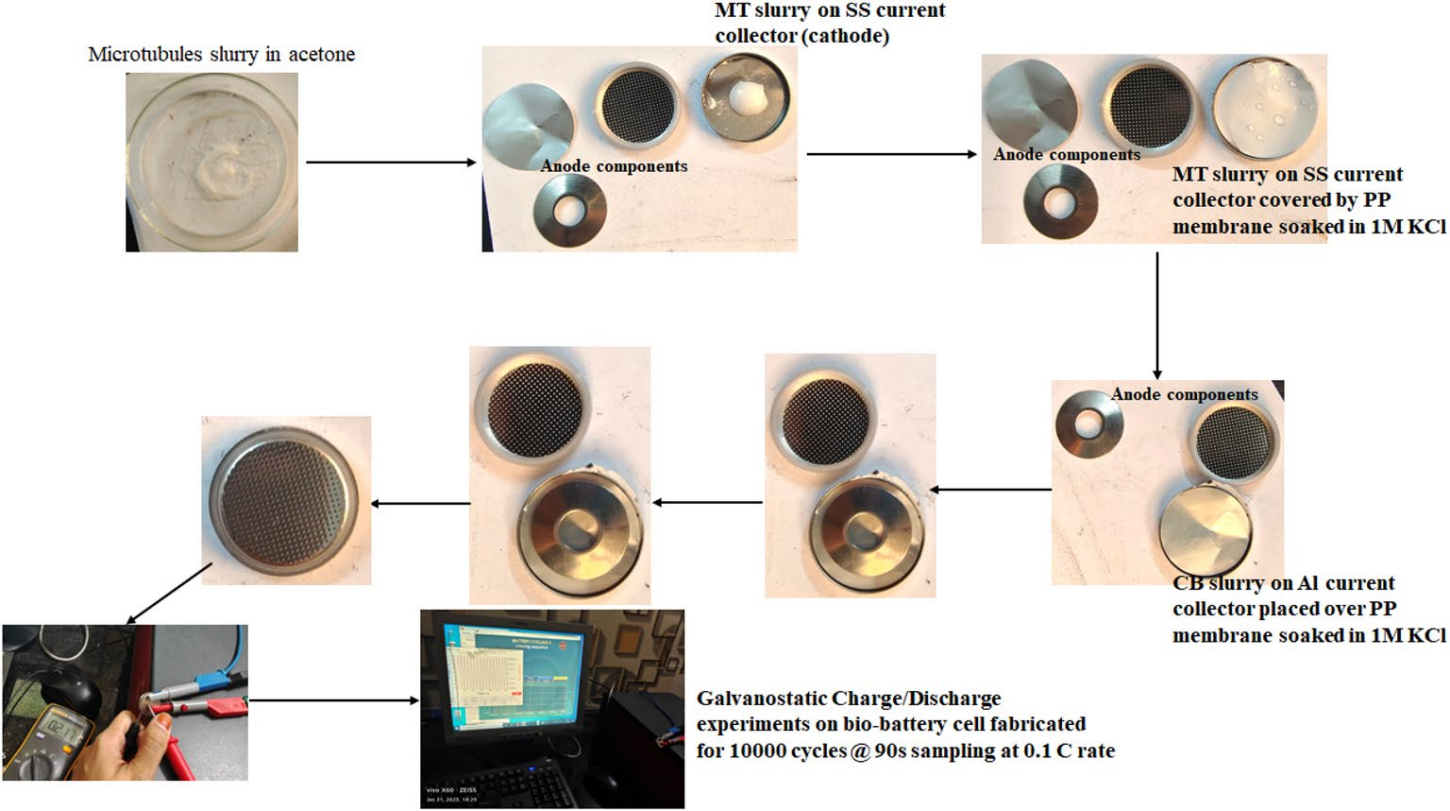
The Galvanostatic Charge/Discharge profile of the fabricated 2032 coin cell of the bio-battery (SS as cathode current collector, MT cathode, PP membrane soaked in 1 mol/L KCl electrolyte, Carbon Black anode, Al anode current collector) Al/CB//PP-1 mol/L KCl/MT/SS.

Thus, at 0.1C rate of discharge, the initial specific capacity and specific capacitance of 63.2 mAh/g and 173.65 F/g respectively during discharge with Δt = 90 s, I = 1A, m = 0.5 g, ΔV = 0.214 V. The subsequent cycles show slight variation in the capacity and capacitance leading to steady decrement at every cycle and leveled at 36.35 mAh/g and 101.33 F/g after 10,000 cycles. This corresponds to 42.48% capacity retention after 10,000 cycles (cf. Figure 18). This demonstrates that the bio-battery cells fabricated in the present invention possess very long cycling stability upto 10,000 cycles at 0.1 C rate and can be utilized in low power electronic devices and power bank applications. The fabricated full cell is shown in Fig. 19.

The energy density of the Bio-Battery cells is calculated employing the equation

$${\text{ED }} = {\text{ Nominal}}\,{\text{ Battery}}\,{\text{ voltage }}*{\text{ Rated}}\,{\text{ Battery }}\,{\text{capacity}}/{\text{Battery}}\,{\text{ weight }} = {\text{ V}}*{\text{C}}/{\text{m}}$$

For 1 kg battery, with nominal voltage of 0.214 V and rated battery capacity being 63.2 mAh/g, the gravimetric energy density is obtained as 13.53 Wh/kg attributed to the protein type Microtubules (MTs) cathode and Carbon Black anode with the full cell configuration of **Al/CB//PP-1MKCl//MTs/SS**. The bio-battery cell of second configuration **Al/EC//PP-1MKCl//MTs/SS** possessed much less performance with stability upto 500 cycles with lower specific capacity and gravimetric energy density. Hence of all configurations attempted, the one with carbon-based anode such as carbon black, graphite or graphene possessed good performance as biomolecules-based energy storge devices for low power electronic device charging and power banks.

## Perspectives and summary

This study will pave way to understand the utility of MTs, synthetic MTs and other similar compounds in bioelectronic applications such as biodegradable batteries, pacemakers so on and so forth. The estimation of electrical conductivity of MTs in liquid phase becomes an interesting study due to the fact that MTs are involved in the electron and proton transport (i) in the cell for phototaxis process, (ii) in centrioles and (iii) in cells coupled with GTP hydrolysis. In addition, as MTs can act as connectors for electric pulse transmission in axons of nerve cells, current study can help in understanding the physical processes associated with consciousness studies. Moreover, as plant MTs are found to handle electromagnetic signals and electrical signals^5,16,23^, present investigation will be of much utility in the studies of quantum information and signal processing in axons involving MTs. The novelty of the present work lies in the fact of utilization of less computer intensive MC simulation coupled with macro characteristics of solvent to understand and evaluate the free energy and kinetic parameters of MTs. Validating the theory with simple electrochemical techniques to measure electronic conductivity, free energy of activation, electrode kinetics due to charge perturbations so on and so forth. Analogously, from the free energetics of the MT conformation, the conductivity can be estimated as suggested in Butler- Volmer equation. Thus, the process associated with the conformation, energetics and ionic as well as electronic conductivity of MTs can be validated. Thus, from CV studies, the reversible peak at 0.3 V indicated the reversible desorption of MT from Au surface without any degradation. As explained in “Free energy of adsorption of MT on Au surface (ΔG) calculated from CV” section, with increase in the applied load, the reversible tubulin adsorption on Au surface becomes more facile with decrease in the activation energy. The decrement in activation energy with increase of load, is approximately equivalent to free energy of tubulin immobilization^33^ (50 meV) on MTs. Thus, with increase in the charge perturbation, the feasibility of MTs adsorbed on the Au surface increases with very minimal variation with load applied (40 meV). The charge transfer resistance is also found to decrease with increase in applied load and as a consequence, tubulin become more reactive to the electrolyte medium by associating the charged counter ions around them and conducting the positive ions such as K^+^ from bulk to the Au surface. The electronic conductivity (σ) ranges from 0.86 to 3.89 mSm^−1^ (as obtained from CV studies) demonstrating 4.58 times increase in σ for 20 times increase in the load. This supported the fact that proteins can be semiconducting in nature^10^, especially MTs and can be of great importance in bioelectronic applications such as biodegradable batteries, pace makers so on and so forth. In addition, Fig. 7, explicitly depicts the dynamic nature of the MTs formed on Au surface to the self-assembly of tubulin monomers in bulk of the electrolyte upon charge perturbations. MC simulation showed (cf. Figure 11) the load dependency of *E*_*ad*_. As the load applied increases, the *E*_*ad*_ decreased, indicating facile adsorption of monomer units and dimerization of tubulin monomers on the Au surface. From Eq. (16), as L → 0, the energy of dimerization will be 3.0 eV which is in good agreement with the literature reported experimental value of 2.6–3.1 eV by Eley et al.^37^. Thus the present methodology could predict the experimental value of *forbidden band width between the highest filled and the unfilled band calculated in proteins stating the possibility of conduction in proteins like semiconductors*^38–40^. The calculated value of ionic conductance for 1 mM tubulin at 0.4 mol/L KCl is 5.53 × 10^–4^ µS (ionic conductivity being 1.1 S m^−1^) from MC simulation whereas the electronic conductivity obtained being 2.89 mS m^−1^ and is 1.93 times higher than the literature. Thus, the current MC simulation in conjunction with CV studies can predict the dynamic stability of MTs and tubulin monomers on Au surface due to external charge perturbations effectively. From the predictions and experimental validation, it is concluded that MTs extracted from plant sources such as Arachis hypogea can act as a semiconductor material on Au surface in KCl environment. Thus, these studies paved way for the future applications of MTs as electrode materials in bioelectronic applications such as biodegradable batteries, pace makers so on and so forth. With the results of theoretical model and the electrochemical experimental validation, two-coin cells of bio-battery fabricated employing MTs as cathode material and CB in cell 1, EC in cell 2 as anode material, PP soaked in 1 mol/L KCl as membrane and electrolyte respectively. At 0.1C rate of discharge, the initial specific capacity and specific capacitance of 63.2 mAh/g and 173.65 F/g respectively during discharge with Δt = 90 s, I = 1A, m = 0.5 g, ΔV = 0.214 V were noticed for cell 1. The subsequent cycles show slight variation in the capacity and capacitance indicating steady decrement at every cycle and leveled at 36.35 mAh/g and 101.33 F/g after 10,000 cycles. This corresponds to 42.48% capacity retention after 10,000 cycles. This demonstrates that the bio-battery cells fabricated in the present investigation possess very long cycling stability upto 10,000 cycles at 0.1 C rate and can be utilized in low power electronic devices and power bank applications (cf. Fig. 18).

## Supplementary Information

Supplementary Information 1.

Supplementary Information 2.

Supplementary Information 3.

Supplementary Information 4.

Supplementary Information 5.

## Data Availability

All data generated or analysed during this study are included in this published article [and its supplementary information files].

## List of symbols

CV: Cyclic voltammetry
GCD: Galvanostatic charge discharge
CB: Carbon black
MTs: Microtubules
SS: Stainless steel
Al: Aluminium
STP: Standard temperature and pressure
EC: Ethyl cellulose
PP: Polypropylene
MC: Monte carlo
ED: Energy density
D: Diffusion co-efficient
F: Faraday’s constant
R: Gas constant
T: Temperature in K
k: Boltzmann constant in J/K
h: Planck’s constant in Js
µ: Mobility
*i*: Current density in Acm^−2^
G: Conductance of the electrolyte in S
${{\Lambda }^{0}}_{{K}^{+}}$: Molar conductance (mhos) of K^+^
C: Concentration of KCl in the electrolyte mol lit^−1^
A: Area of the cross section of electrodes in cm^2^
l: Distance between the electrodes in cm
*E*_*ad*_: Dimerization energy in eV
µ_1_ and µ_2_: The chemical potential of α and β tubulin in eV
ρ: Resistivity of the dimers in Ohms
σ: Conductivity of the dimers in Sm^−1^
R_ct_: Charge-transfer resistance in Ohms
$\Delta G$: Free energy of adsorption of MT on Au surface in eV
k_et_: Heterogeneous rate constant in ms^−1^

## References

[1] Janke C (2014). The tubulin code: Daltons components, readout mechanisms, and functions. J. Cell Biol..

[2] Li H, DeRosier DJ, Nicholson WV, Nogales E, Downing KH (2002). Microtubule structure at 8 Å resolution. Structure.

[3] Tuszyński JA, Hameroff S, Satarić MV, Trpisova B, Nip MLA (1995). Ferroelectric behavior in microtubule dipole lattices: Implications for information processing, signaling and assembly/disassembly. J. Theor. Biol..

[4] Alushin GM, Lander GC, Kellogg EH, Zhang R, Baker D, Nogales E (2014). High-resolution microtubule structures reveal the structural transitions in αβ-Tubulin upon GTP hydrolysis. Cell.

[5] Tuszynski JA, Makarov S, Horner M, Noetscher G (2019). The bioelectric circuitry of the cell. 2019 Aug 28. Brain and Human Body Modeling: Computational Human Modeling at EMBC 2018 [Internet].

[6] Minoura I, Muto E (2006). Dielectric measurement of individual microtubules using the electroorientation method. Biophys. J..

[7] Van den Heuvel M, De Graaff M, Lemay S, Dekker C (2007). Electrophoresis of individual microtubules in microchannels. Proc. Natl. Acad. Sci..

[8] Kirson ED, Dbaly V, Tovarys F, Vymazal J, Soustiel JF, Itzhaki A (2007). Alternating electric fields arrest cell proliferation in animal tumor models and human brain tumors. Proc. Natl. Acad. Sci..

[9] Tuszynski J, Wenger C, Friesen D, Preto J (2016). An overview of sub-cellular mechanisms involved in the action of TTFields. Int. J. Environ. Res. Public Health.

[10] Rosenberg B (1962). Electrical conductivity of proteins. Nature.

[11] Santelices IB, Douglas EF, Clayton B, Cameron MH, Jack X, Aarat K, Piyush K (2017). Response to alternating electric fields of tubulin dimers and microtubule ensembles in electrolytic solutions. Sci. Rep..

[12] Mitchison T, Marc K (1984). Dynamic instability of microtubule growth. Nature.

[13] Craddock TJA, Jack AT (2007). On the role of the microtubules in cognitive brain functions. NeuroQuantology.

[14] Gundersen GG, Cook TA (1999). Microtubules and signal transduction. Curr. Opin. Cell Biol..

[15] Hirokawa N (1998). Kinesin and dynein superfamily proteins and the mechanism of organelle transport. Science.

[16] Cantero MDR, Villa Etchegoyen C, Perez PL, Scarinci N, Cantiello HF (2018). Bundles of brain microtubules generate electrical oscillations. Sci. Rep..

[17] Barlow PW (2015). The natural history of consciousness, and the question of whether plants are conscious, in relation to the Hameroff-Penrose quantum-physical ‘Orch OR’theory of universal consciousness. Commun. Integr. Biol..

[18] Stuart H (1998). Quantum computation in brain microtubules? The Penrose-Hameroff ‘Orch OR’ model of consciousness. Philos. Trans. R. Soc. Lond. Ser. A Math. Phys. Eng. Sci..

[19] Hameroff S, Penrose R (1996). Orchestrated reduction of quantum coherence in brain microtubules: A model for consciousness. Math. Comput. Simul..

[20] Craddock TJA, Jack AT, Avner P, Holly F (2010). Microtubule ionic conduction and its implications for higher cognitive functions. J. Integr. Neurosci..

[21] Sekulić D, Satarić MV (2014). An improved nanoscale transmission line model of microtubule: The effect of nonlinearity on the propagation of electrical signals. Facta Univ. Ser. Electron. Energetics.

[22] Frieden BR, Gatenby RA (2019). Signal transmission through elements of the cytoskeleton forms an optimized information network in eukaryotic cells. Sci. Rep..

[23] Dent EW, Baas PW (2014). Microtubules in neurons as information carriers. J. Neurochem..

[24] Freedman H, Rezania V, Priel A, Carpenter E, Noskov SY, Tuszynski JA (2010). Model of ionic currents through microtubule nanopores and the lumen. Phys. Rev. E.

[25] Satarić MV, Sekulić D, Živanov M (2010). Solitonic ionic currents along microtubules. J. Comput. Theor. Nanosci..

[26] Umnov M, Palusinski OA, Pierre AD, Guzman R, Hoying J, Hugh Barnaby YY, Srini R (2007). Experimental evaluation of electrical conductivity of microtubules. J. Mater. Sci..

[27] Kononova O, Kholodov Y, Theisen KE, Marx KA, Dima RI, Ataullakhanov FI, Grishchuk EL, Barsegov V (2014). Tubulin bond energies and microtubule biomechanics determined from nanoindentation in silico. J. Am. Chem. Soc..

[28] Detrich HW, Johnson KA, Marchese-Ragona SP (1989). Polymerization of Antarctic fish tubulins at low temperatures: Energetic aspects. Biochemistry.

[29] Padmapriya S, Harinipriya S (2019). Hydrogen storage capacity of polypyrrole in alkaline medium: Effect of oxidants and counter anions. J. Market. Res..

[30] Harinipriya S, Aarat K, Amit KM (2016). Physiochemical characterization of tubulin from *Arachis hypogaea*. Synth. Met..

[31] Sukeri A, Bertotti M (2018). Nanoporous gold surface: An efficient platform for hydrogen evolution reaction at very low overpotential. J. Braz. Chem. Soc..

[32] Hoefling M, Iori F, Corni S, Gottschalk KE (2010). Interaction of amino acids with the Au (111) surface: Adsorption free energies from molecular dynamics simulations. Langmuir.

[33] Mozziconacci J, Sandblad L, Wachsmuth M, Brunner D, Karsenti E (2008). Tubulin dimers oligomerize before their incorporation into microtubules. PLoS ONE.

[34] Bard A, Falkner LR (1990). Electrochemical Methods.

[35] Sept D, Baker NA, McCammon JA (2003). The physical basis of microtubule structure and stability. Protein Sci..

[36] Bürgi T (2015). Properties of the gold–sulphur interface: From self-assembled monolayers to clusters. Nanoscale.

[37] Cardew MH, Eley DD (1959). The semiconductivity of organic substances. Part 3.—Haemoglobin and some amino acids. Discuss. Faraday Soc..

[38] Ladik J (1964). Energy band structure of proteins. Nature.

[39] Rosenberg B, Postow E (1969). Semiconduction in proteins and lipids—Its possible biological import. Ann. N. Y. Acad. Sci..

[40] Evans MG, Gergely J (1949). A discussion of the possibility of bands of energy levels in proteins electronic interaction in non-bonded systems. Biochem. Biophys. Acta..

[41] Ayoub AT, Craddock TJ, Klobukowski M, Tuszynski J (2014). Analysis of the strength of interfacial hydrogen bonds between tubulin dimers using quantum theory of atoms in molecules. Biophys. J..

[42] Shen C, Guo W (2018). Ion permeability of a microtubule in neuron environment. J. Phys. Chem. Lett..

[43] Craddock TJ, Tuszynski JA, Priel A, Freedman H (2010). Microtubule ionic conduction and its implications for higher cognitive functions. J. Integr. Neurosci..

[44] McIntosh JR, Morphew MK, Grissom PM, Gilbert SP, Hoenger A (2009). Lattice structure of cytoplasmic microtubules in a cultured Mammalian cell. J. Mol. Biol..

[45] Benson GC, Gordon AR (1945). A reinvestigation of the conductance of aqueous solutions of potassium chloride, sodium chloride, and potassium bromide at temperatures from 15 to 45 °C. J. Chem. Phys..

[46] Krouglova T, Vercammen J, Engelborghs Y (2004). Correct diffusion coefficients of proteins in fluorescence correlation spectroscopy. Application to tubulin oligomers induced by Mg2+ and Paclitaxel. Biophys. J..

[47] Stracke R, Böhm KJ, Wollweber L, Tuszynski JA, Unger E (2002). Analysis of the migration behaviour of single microtubules in electric fields. Biochem. Biophys. Res. Commun..

